# Histologic and Endoscopic Similarity between Nodular Gastric Antral Vascular Ectasia and Gastric Hyperplastic Polyps Potentially Causing Treatment Delays

**DOI:** 10.1155/2019/1342368

**Published:** 2019-09-29

**Authors:** Pujitha Kudaravalli, Sheikh A. Saleem, Rachana Mandru, Sekou Rawlins

**Affiliations:** ^1^Department of Internal Medicine, Upstate Medical University, 750 E. Adams Street, Syracuse, NY 13210, USA; ^2^Department of Gastroenterology, Upstate Medical University, 750 E. Adams Street, Syracuse, NY 13210, USA

## Abstract

**Introduction:**

Gastric antral vascular ectasia (GAVE) is the underlying cause for 4% of nonvariceal upper GI bleeding. Nodular GAVE and gastric hyperplastic polyps have similar appearance on upper GI endoscopy (EGD) as well as histology, which could delay specific targeted therapy. We herein, through this case, would like to highlight that high clinical suspicion is required to diagnose nodular GAVE.

**Case Report:**

A 70-year-old male with a past medical history significant for coronary artery disease s/p drug-eluting stent placement on Plavix, coronary artery bypass grafting, mechanical aortic valve replacement on warfarin, and iron deficiency anemia on replacement was admitted for the evaluation of fatigue and melena for a month. Physical examination was positive for black stool. The only significant lab was a drop in hemoglobin/hematocrit (Hg/dl/H%) of 10/32 to 4/12.5. Fibrosure was sought which suggested that the patient had an F4 cirrhosis. Endoscopy showed nodules in the gastric antrum which were presumptively treated as GAVE with argon plasma coagulation (APC). Surgical pathology showed reactive gastropathy and gastric polyps. Review of the past histology suggested that because of the overlap in the histopathological features of hyperplastic polyps and GAVE, they were misinterpreted as hyperplastic polyp rather than nodular GAVE.

**Discussion:**

GAVE can be classified endoscopically as punctate, striped, nodular, or polypoidal form. The light microscopic findings considered specific to GAVE are vascular hyperplasia, mucosal vascular ectasia, intravascular fibrin thrombi, and fibromuscular hyperplasia. However, these findings do not differentiate GAVE from hyperplastic gastric polyp. The first line of treatment for GAVE is endoscopic ablation with Nd:YAG laser or argon plasma coagulation. Response to therapy was seen with a mean of 2.6 treatment sessions. There is not a lot of evidence supportive of pharmacological treatment of GAVE with estrogen-progesterone, tranexamic acid, and thalidomide. Serial endoscopic band ligation as well as detachable snares in the management of nodular GAVE refractory to argon plasma coagulation has also been tried.

**Conclusion:**

Oftentimes, there is a delay in the diagnosis and treatment of nodular GAVE as the histopathological appearance could be similar to gastric polyps. The diagnosis of GAVE especially nodular GAVE requires a high level of clinical suspicion. Misdiagnosis of nodular GAVE can delay targeted therapy and have fatal outcomes.

## 1. Introduction

Gastric antral vascular ectasia (GAVE) is the underlying cause for 4% of nonvariceal upper gastrointestinal bleeding [1]. GAVE is characterized by tortuous, dilated vessels in the gastric antrum. Endoscopically, it has a characteristic flat, striped erythematous appearance involving the gastric antrum, but it could also present as nodules [2].

Nodular GAVE and gastric hyperplastic polyps have similar appearance on endoscopy as well as histology [3]. We herein, present a case of a 70-year-old male who was found to have nodules in the gastric antrum, which based on their endoscopic appearance and histological features were initially diagnosed as gastric hyperplastic polyps but were later confirmed as nodular GAVE.

## 2. Case Report

A 70-year-old male with multiple medical conditions was admitted to the hospital for evaluation of acute on chronic iron deficiency anemia. Blood work at the patient's outpatient visit in February 2018 showed a significant decline in his baseline hemoglobin level of ∼10–11 g/dl to a hemoglobin level of 4 g/dl with a hematocrit level of 12.5%. The patient endorsed melena, fatigue, and generalized weakness for a month. He denied nausea, vomiting, abdominal pain, fever, chills, or changes in his bowel habits. The patient's past medical history was significant for hypertension, dyslipidemia, type II diabetes mellitus, coronary artery disease, and non-ST segment elevation myocardial infarction in 11/2017 s/p saphenous venous graft revascularization with DES. Past surgical history included coronary artery bypass graft (CABG), along with mechanical aortic valve replacement in 2005, right hemicolectomy for advanced adenoma in 2000, and abdominal aortic aneurysm repair. His home medications included Plavix, warfarin, pantoprazole, ferrous sulphate, vitamin C, vitamin B12, torsemide, glipizide, metformin, and atorvastatin. He quit smoking 26 years ago and denied the use of alcohol or any illicit drugs.

On examination, he was well developed and oriented to time, place, and person. He was hemodynamically stable. Abdominal, cardiovascular, and respiratory system examination was unremarkable. On rectal examination, he had black stool and external hemorrhoids.

**Figure 1:**
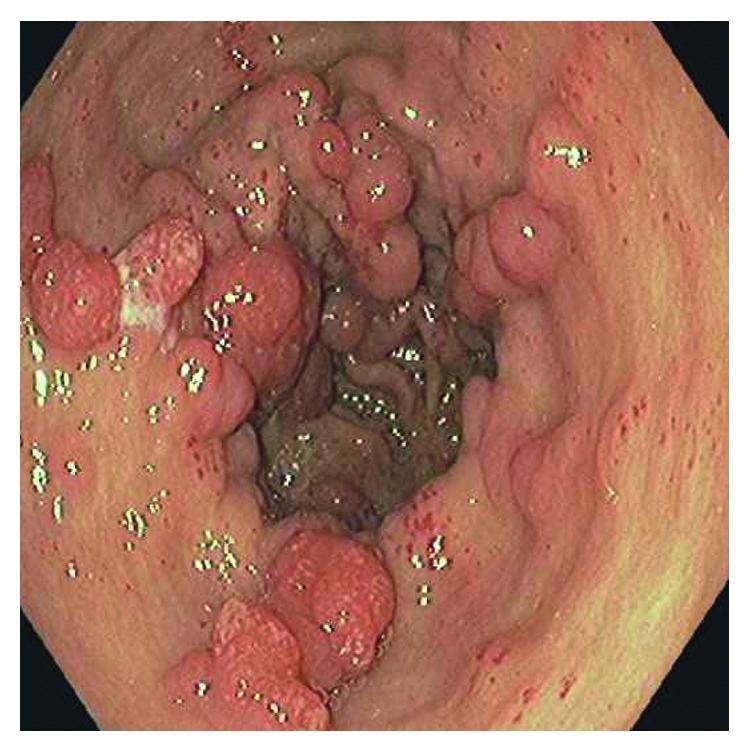
Columns of tortuous ectatic vessels in the gastric antrum that appear similar to hyperplastic polyps.

**Figure 2:**
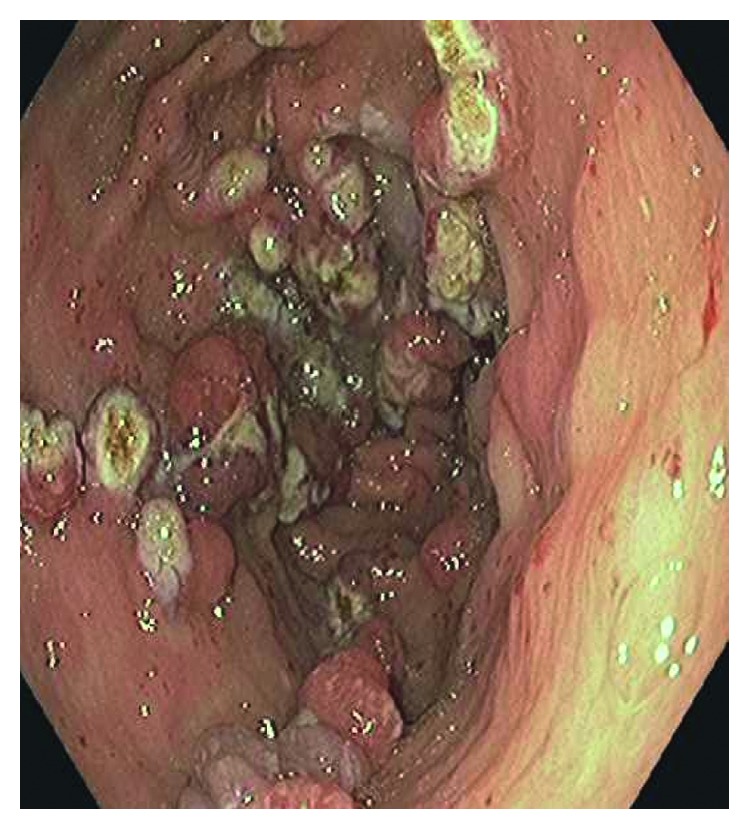
Nodular GAVE after treating with argon plasma coagulation (APC).

His platelet count was 250,000 *μ*L, WBC count was 5000 *μ*L, INR was 1.58, BUN was 14 mg/dl, and creatinine was 0.67 mg/dl on admission. Upper gastrointestinal endoscopy was done which showed nodules in the gastric antrum which were presumptively treated as GAVE with argon plasma coagulation (APC) (Figures 1 and 2). Biopsy was taken during the endoscopy, and the surgical pathological reading was reported as reactive gastropathy and gastric polyps. In retrospect, the patient had similar episodes of melena in 2015 when the gastric antrum nodules were reported as gastric polyps. Biopsy of the polyps in 2015 was interpreted as hyperplastic polyp.

Fibrosure was sought which suggested that the patient had an F4 fibrosis. The presence of chronic iron deficiency anemia, melena, cirrhosis, and nodular appearance with vascular ectasia in the antral region on endoscopy lead to the diagnosis of nodular GAVE in our patient despite the absence of histopathological confirmation. The patient responded to APC, and the hemoglobin level at discharge was 10 g/dl and the Hct level was 31.6%. The patient continued to maintain Hb > 7 and received a second session of APC in June 2018. Review of the past literature suggested that because of the overlap in the histopathological features of hyperplastic polyps and GAVE, they were misinterpreted as hyperplastic polyp rather than nodular GAVE.

## 3. Discussion

Gastric antral vascular ectasia (GAVE) was first described by Rider et al. in 1953, and it was only years later the term watermelon stomach was coined by Jabbari et al. in 1984. GAVE is classified endoscopically as punctate-type and striped-type lesions [3]. GAVE can also be classified endoscopically as flat punctate, flat striped pattern, nodular, or polypoidal form [2]. All patients with punctate-type vascular ectasias have cirrhosis and only 38% of those with striped pattern have cirrhosis [3]. Some of the diseases associated with GAVE are scleroderma, mixed connective disease, history of cancer, cryptogenic cirrhosis, and chronic renal failure. The prototypic patient is usually a female with an average age of 73 [4].

The pathogenesis of GAVE remains unclear. One proposed hypothesis is that the mechanical stress with repeated prolapse of antral mucosa by forceful peristalsis results in obstruction of blood flow to the mucosa and submucosa resulting in ectasia [2]. The abnormal antral motility seen in cirrhosis causes mechanical stress, and this could contribute in explaining the association between cirrhosis and GAVE [5].

Presence of portal hypertension does not contribute to the development of GAVE. A study done by Vincent et al. showed that GAVE disappeared after liver transplantation despite persistence of portal hypertension after surgery [6]. It is also important to differentiate portal hypertensive gastropathy (PHG) from GAVE as the treatment modality differs. PHG affects the fundus or corpus of the stomach and has a mosaic-like or snake skin pattern. GAVE affects the antrum of the stomach and occasionally even the proximal part of the stomach. Treatment of portal hypertension resolves PHG but not GAVE.

The light microscopic findings [7] considered specific to GAVE are vascular hyperplasia, mucosal vascular ectasia, intravascular fibrin thrombi, and fibromuscular hyperplasia. However, these findings do not differentiate GAVE from hyperplastic gastric polyp.

The first line of treatment for GAVE is endoscopic ablation with Nd:YAG laser or argon plasma coagulation (APC). Response to therapy was seen with a mean of 2.6 treatment sessions [8]. There is not a lot of evidence supportive of pharmacological treatment of GAVE with estrogen-progesterone, tranexamic acid, and thalidomide, which now have been used only if endoscopic ablation showed no response. Serial endoscopic band ligation as well as detachable snares in the management of nodular GAVE refractory to APC has also been tried. In a case report by Wright et al., a patient's anemia and GI blood loss resolved after multiple sessions of band ligation [9].

## 4. Conclusion

Oftentimes, there is a delay in the diagnosis and treatment of nodular GAVE as the histopathological appearance could be similar to gastric polyps. The diagnosis of GAVE, especially nodular GAVE, requires a high level of clinical suspicion. Misdiagnosis of nodular GAVE can delay targeted therapy and have fatal outcomes.
